# Cortisol levels and depression suicide risk: a combined exploration of meta-analysis and case-control study

**DOI:** 10.3389/fpsyt.2025.1563819

**Published:** 2025-04-30

**Authors:** Zhaowei Jiang, Liying Dong, Yajie Zhang, Hongjing Mao, Fugang Luo, Mingfen Song

**Affiliations:** ^1^ The Fourth Clinical Medical College of Zhejiang Chinese Medical University, Zhejiang Chinese Medical University, Hangzhou, China; ^2^ Department of Psychiatry, Shaoxing Seventh People's Hospital, Shaoxing, China; ^3^ Affiliated Mental Health Center and Hangzhou Seventh People's Hospital, Zhejiang University School of Medicine, Hangzhou, China

**Keywords:** cortisol, hair cortisol, depression, suicide, meta

## Abstract

**Objective:**

To evaluate the association between cortisol levels and suicide in patients with depression through a meta-analysis to provide an early warning for suicide prevention.

**Methods:**

Databases including China National Knowledge Infrastructure (CNKI), Weipu Database (VIP), Wanfang Database, PubMed, Cochrane Library, Web of Science, and Embase were searched to collect case-control studies, cohort studies, and cross-sectional studies investigating the relationship between cortisol levels and suicide in patients with depression. The meta-analysis was conducted using Stata 18.0. Meanwhile, we collected data from 131 participants to measure their cortisol levels, including Hair cortisol levels (HCL) were calculated for the 0–1 cm and 1–2 cm segments.

**Results:**

The meta-analysis indicated that cortisol levels in depressed patients with suicidal behavior were significantly higher than healthy individuals (SMD = 0.350, 95% CI [0.003, 0.696]). However, the cortisol levels in patients with suicidal behavior were only slightly higher than those in patients without suicidal behavior, and the pooled effect size (SMD = 0.108, 95% CI [-0.151, 0.367]) was non-significant. The depression patients with suicidal behaviors had significantly lower levels of 0–1 cm HCL (3.46 ± 1.92 ng/mg) than those in the depression patients without suicidal ideation (5.43 ± 2.42 ng/mg) (P=0.003) and in the depression patients with suicidal ideation (5.04 ± 2.30 ng/mg) (P=0.037). Similarly, 1–2 cm HCL was significantly lower in the depression with suicidal behavior group (3.21 ± 1.47 ng/mg) than in the depression without suicidal ideation group (5.65 ± 3.59 ng/mg) (P=0.009) and in the depression with suicidal ideation group (5.10 ± 2.88 ng/mg) (P=0.050).

**Conclusion:**

The study found that cortisol levels in patients with depression who exhibited suicidal behavior were higher than those in the healthy population. However, no significant difference in cortisol levels was observed between patients with depression and suicidal behavior and those without suicidal behavior. The experiment revealed that patients with depression and suicidal behavior had lower hair cortisol levels (HCL) than those depression without suicidal behavior.

**Systematic Review Registration:**

https://www.crd.york.ac.uk/PROSPERO/view/CRD42024609538 **, identifier CRD42024609538.**

## 1 Introduction

According to the World Health Organization (WHO), as of 2021, suicide ranks as the third leading cause of death among individuals aged 15–29 years globally, with over 720,000 deaths by suicide annually and many more suicide attempts (1). In high-income countries, mental disorders, particularly depression, are strongly associated with suicide and previous suicide attempts. Globally, approximately 3.8% of the population suffer from depression, including 5% of adults (4% men, 6% female) and 5.7% of adults aged 60 years and older (2). In the United States, approximately 20% of adults have a lifetime diagnosis of major depressive disorder (MDD) (3).

Suicidal behavior is generally defined as potentially lethal actions undertaken with the intent of ending one’s life. It is further categorized into three distinct types: suicide, suicide attempts, and suicidal ideation (4, 5). A meta-analysis estimated that the lifetime prevalence of suicide attempts in individuals with MDD is 31% (6).

Numerous studies have highlighted the association between depressive disorders and dysregulation of the hypothalamic-pituitary-adrenal (HPA) axis (7). Like other endocrine systems, the HPA axis operates through a negative feedback regulatory mechanism, where hypothalamic and pituitary receptors detect circulating cortisol levels. Elevated cortisol levels inhibit secretion, whereas reduced levels stimulate it. However, chronic HPA axis activation may lead to cortisol hypersecretion, resulting in prolonged tissue exposure to elevated cortisol concentrations (8). Cross-sectional studies indicate that MDD patients exhibit higher cortisol levels compared to healthy controls (9, 10). Prospective studies further reveal that elevated cortisol predicts subsequent MDD onset, suggesting HPA axis dysfunction may precede depressive episodes independently of age (11). Cortisol levels fluctuated according to the circadian rhythm of the HPA axis, peaking in the early morning and declining in the evening. Depressive symptoms often induce chronic stress in patients, which disrupts various endocrine functions. Negative emotions and poor sleep quality can lead to HPA axis dysregulation, which results in elevated cortisol levels. When encountering stress, the HPA axis is activated, triggering the release of cortisol from the adrenal glands. Cortisol has been shown to enhance energy mobilization and protein and lipid breakdown, and regulate the magnitude and duration of inflammatory responses (12). These mechanisms have also been implicated in suicidal behavior (13).

Current evidence reveals complex cortisol-suicidality relationships: Elevated cortisol may increase suicide risk by impairing prefrontal cortex (PFC) function - a brain region critical for impulse control, decision-making, and emotional regulation (9). For instance, suicide attempters demonstrate abnormal stress-induced cortisol responses correlated with impulsivity traits (14). Conversely, chronic HPA axis hyperactivity may induce glucocorticoid receptor desensitization (15), ultimately leading to cortisol depletion. This hypocortisolism may correspond to physiological compensation during post-attempt recovery phases (16). Current consensus identifies glucocorticoid resistance (impaired receptor-mediated feedback) as the central mechanism underlying HPA axis dysregulation and cortisol elevation in MDD (17, 18). Genetic polymorphisms in HPA axis-related genes may predispose individuals to cortisol abnormalities and increased suicide risk (19, 20). Epigenetic mechanisms like DNA methylation mediate environmental effects on cortisol dysregulation and suicidality (20).

While some studies report elevated hair cortisol concentrations in suicide attempters, with higher levels in multiple attempters (21), contradictory findings exist. One adolescent suicidality study found an inverse relationship between attempt frequency and serum cortisol (22). Sex- and age-specific patterns emerge: Female MDD patients exhibit higher hair cortisol concentrations and cortisol awakening responses (CAR), but blunted stress reactivity (23). Depressed women show attenuated morning cortisol and flattened diurnal slopes (reduced peak amplitude and diminished nocturnal decline), particularly during pregnancy (24, 25). Adolescents demonstrate exaggerated cortisol fluctuations under stress (26), adults exhibit variable stress recovery kinetics (27), while elderly populations display flattened cortisol curves (28). A critical age-dependent reversal was observed: positive cortisol-suicidality correlation before age 40 *vs*. negative correlation post-40 (29).

To reconcile conflicting evidence, our systematic review compared cortisol profiles between suicidal and non-suicidal MDD patients. While MDD patients generally exhibit hypercortisolism versus healthy controls, morning cortisol declines may reflect chronic stress adaptation or axis fatigue (30). Notably, suicidal MDD patients demonstrate lower cortisol levels than other psychiatric patients - potentially due to enhanced negative feedback or reduced biosynthesis - despite higher morning cortisol (30). Current limitations include: Predominance of cross-sectional designs over longitudinal studies (single-timepoint measurements cannot characterize temporal dynamics). Heterogeneous sampling across age/gender groups and sociocultural contexts (variations in suicide perception and stress coping) (31). Methodological variability in biofluid collection (blood/saliva = acute levels *vs*. hair = chronic exposure) (32). Analytical differences across measurement platforms (ELISA, CLIA, LC-MS/MS) with varying sensitivity and specificity (33).

Our updated meta-analysis aimed to explore the relationship between cortisol levels in patients with depression exhibiting suicidal behavior, those without suicidal behavior, and healthy controls. This study was registered on the PROSPERO website, identifier CRD42024609538.

## 2 Materials and methods

### 2.1 Inclusion criteria

1) Study Design: Cross-sectional, cohort, and case-control studies that categorized participants into three groups: depression with suicidal behavior, depression without suicidal behavior, and healthy controls. 2) Study Population: Patients diagnosed with depression, with or without suicidal behavior (including suicide, suicide attempts, or suicidal ideation), or healthy controls. Cortisol measurements included serum, plasma, urinary free, and salivary cortisol levels. 3) Data Availability: Studies providing accessible data for analysis.

### 2.2 Exclusion criteria

1) Studies lacking sufficient data to calculate the mean and standard deviation (SD) of cortisol levels. 2) Meta-analyses, systematic reviews, case reports, letters, conference abstracts, and animal studies. 3) Duplicate publications. 4) Studies without a diagnosis of depression or cortisol data for comparison.

### 2.3 Literature search strategy

We systematically searched the following databases: PubMed, Cochrane Library, Web of Science, Embase, China National Knowledge Infrastructure (CNKI), Weipu Database (VIP), and Wanfang Database. The search aimed to identify case-control, cohort, and cross-sectional studies on the relationship between cortisol levels and suicide in patients with depression. The search period covered all publications from database inception to September 9, 2024. Search Terms: Depression, Depressive Disorder, Depression, Postpartum, Depressive Disorder, Treatment-Resistant, Depressive Disorder, Major, Vascular Depression, Hydrocortisone, Cortisol, Suicide.

### 2.4 Literature screening and data extraction

Two researchers independently screened the literature, extracted the data, and cross-checked the results. In case of disagreement, a third-party evaluator was consulted for arbitration. During the screening process, titles and abstracts were initially reviewed to exclude irrelevant studies, followed by a full-text review to determine the final inclusion based on inclusion and exclusion criteria. The meta-analysis was conducted using Stata 18.0. The extracted data included the first author’s name, publication year, country, study design, population, sample size, age, sex, type of cortisol measurement, and the mean and standard deviation of cortisol levels. Unit conversion for cortisol was performed based on its molecular weight (362.46 g/mol) using the formula: 1 μg/dL=0.0276 nmol/L.

### 2.5 Risk of bias assessment

Observational Studies: The risk of bias in observational studies (case-control and cohort studies) was assessed using the Newcastle-Ottawa Scale (NOS). The scale evaluates three domains: selection of participants (four items), comparability of groups (two items), and exposure or outcome determination (three items). Each criterion fulfilled received one point, with a maximum total score of 9. Studies scoring ≥7 points were considered high-quality (34).

Cross-sectional Studies: The risk of bias in cross-sectional studies was evaluated using the Agency for Healthcare Research and Quality (AHRQ) checklist, which includes 11 items. Responses were recorded as “Yes,” “No,” or “Unclear.” The checklist includes the following criteria: 1) Defining the source of information (survey and record review). 2) List of inclusion and exclusion criteria for exposed and unexposed subjects (cases and controls) or references to previous publications. 3) Indicate the time used to identify the patients. 4) Indicate whether subjects were consecutive, if not population-based. 5) Indicates whether evaluators of subjective components of the study were masked to other aspects of the status of the participants. 6) Describe any assessments undertaken for quality assurance purposes (e.g., test/retest of primary outcome measurements). 7) Explain any patients excluded from the analysis. 8) Describe the assessment and/or control of confounding factors 9) If applicable, explain how missing data were handled during the analysis. 10) Summarize the patient response rates and completeness of data collection. 11) Clarify what follow-up, if any, was expected and the percentage of patients for whom incomplete data or follow-up were obtained (35). The article quality was assessed as follows: low quality = 0-3, moderate quality = 4-7, and high quality = 8-11 (36).

### 2.6 Inclusion and exclusion criteria

This case-control study involved patients diagnosed with depression and matched healthy controls who visited the Hospital between August 2023 and January 2024. The psychological state of having the intent to terminate one’s life through suicidal behavior was defined as suicidal ideation. When individuals not only had suicidal ideation but also performed actions aimed at ending their lives, this was defined as suicidal behavior. This study was approved by the Hospital Ethics Committee, with ethical approval number No. 037, July 2023.

A total of 131 participants were included in the study based on the following inclusion and exclusion criteria: healthy control group, 32 participants; depression without suicidal ideation group, 32 participants; depression with suicidal ideation group, 34 participants; and depression with suicidal behavior group, 33 participants.

### 2.7 Inclusion criteria

Healthy Control Group: 1) No severe physical illness. 2) No suicidal ideation or behavior in the past three months. 3) Aged 16–60 years. 4) HAMD-24 score < 8. 5) Treatment history matched with other groups. Depression without Suicidal Ideation Group: 1) no severe physical illness. 2) No suicidal ideation or behavior in the past month. 3) Aged 16–60 years. 4) HAMD-24 score ≥ 20. 5) Treatment history is not limited. Depression with Suicidal Ideation Group: 1) no severe physical illness. 2) Suicidal ideation present but no suicidal behavior in the past month. 3) Aged 16–60 years. 4) HAMD-24 score ≥ 20. 5) Treatment history is not limited. Depression with Suicidal Behavior Group: 1) no severe physical illness. 2) Suicidal ideation and suicidal behavior present in the past month. 3) Aged 16–60 years. 4) HAMD-24 score ≥ 20. 5) Treatment history is not limited.

### 2.8 Exclusion criteria

1) pregnant or breastfeeding women, or those with irregular menstrual cycles (cycle < 21 days or > 35 days). 2) Individuals recently using hormonal medications or diagnosed with endocrine disorders. 3) Individuals with severe physical illnesses. 4) Depression caused by substance use. 5) Patients with depression due to other psychiatric disorders, such as post-schizophrenia depression or depressive episodes in bipolar disorder.

### 2.9 Data collection and scale assessment

1. Basic Data Collection: Data were collected on participants’ demographic and lifestyle information, including Demographics: Age, sex, height, weight, marital status, occupation, educational background, and Body Mass Index (BMI). Lifestyle Information: Smoking and drinking habits, physical health status, history of mental illness, and family health history. Participants were also asked whether they had experienced stressful events in the past three months. Definitions: Smoking history: Current smokers or individuals who quit smoking less than three years ago. Drinking history: alcohol consumption of ≥40 g/day. Stressful events: Significant life events that induce intense physiological and psychological stress. Examples include bereavement, income loss/unemployment, debt/bankruptcy, etc.
2. Scale Assessment: The 24-item Hamilton Depression Rating Scale (HAMD) was used to evaluate depressive symptoms. Developed by Hamilton in 1960, it is one of the most widely used tools for assessing depressive states in the clinical setting. Assessments were conducted at baseline by two fixed clinicians who underwent standardized training to ensure consistency.
3. Experimental Reagents and Instruments: Cortisol Measurement: Human cortisol enzyme-linked immunosorbent assay (ELISA) kit, 96 wells (Shanghai Enzyme-linked Biotechnology Co., Ltd., Product No.: 202402062115-65A). Plate Reader: Microplate Reader (Shenzhen Rayto Life Sciences Co., Ltd., Rayto RT-6100).

## 3 Results

### 3.1 Literature screening process and results

**Figure 1:**
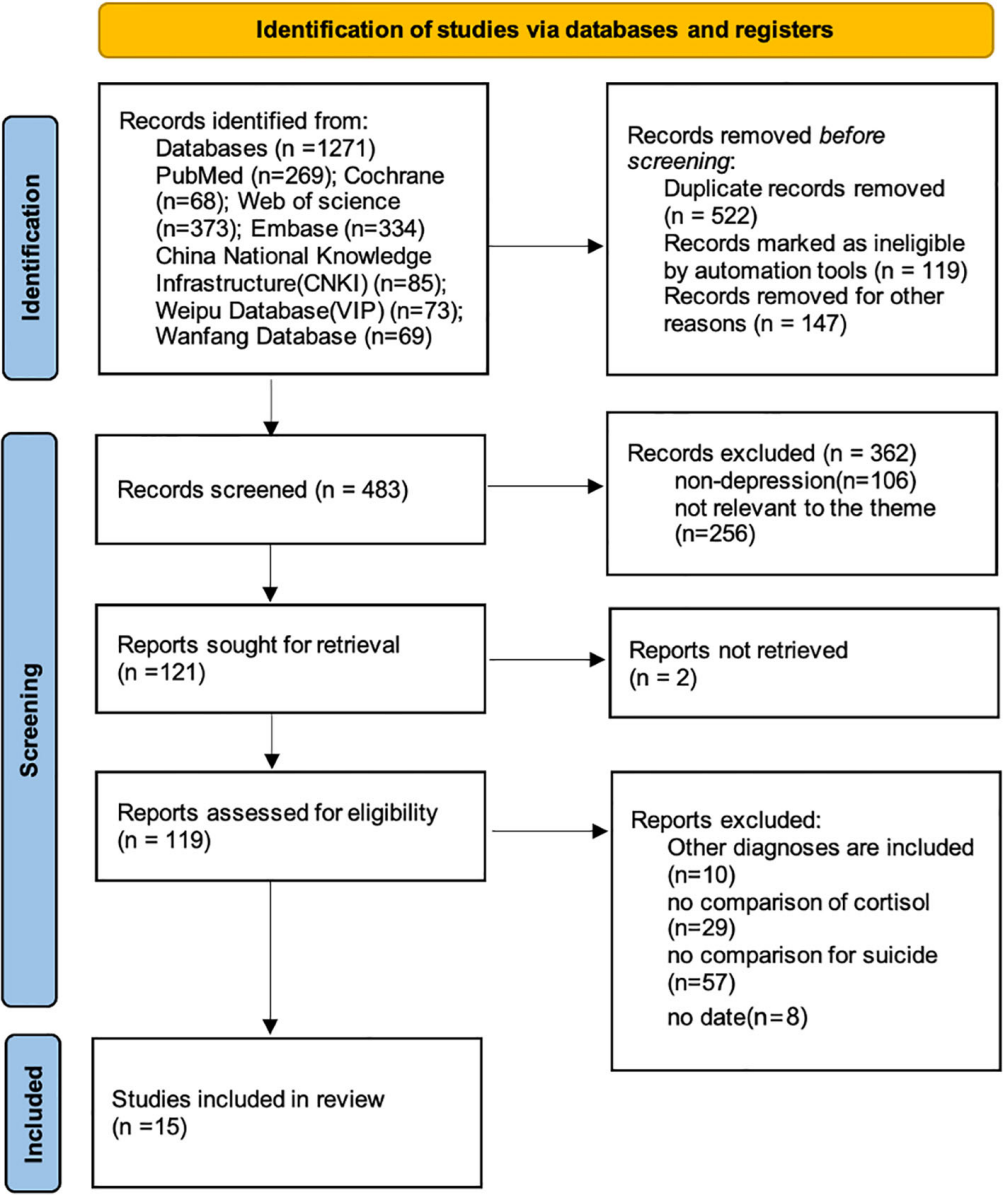
PRISMA flow diagram for studies retrieved through the online search and selection process.

A total of 1,271 studies were identified using the search strategy. After removing 522 duplicate records using EndNote, the remaining studies underwent title and abstract screening followed by a full-text review. Ultimately, 15 studies that met the inclusion criteria were selected for the analysis (21, 37–50). The inclusion criteria were stringent and focused exclusively on studies with a confirmed diagnosis of depressive disorder. The detailed literature screening process is shown in **Figure 1**.

### 3.2 Characteristics of included studies and risk of bias assessment

The quality of included studies was assessed using the AHRQ and NOS scales, as summarized in the general characteristics table (**Table 1**). Despite the variability in quality scores (ranging from 7 to 9 stars), all studies were deemed to be of good quality.

**Table 1:** General characteristics table.

									Cortisol nmol/L									
Author	Year	Country	Age	Sample	Females	Type of cortisol sample	Sample time	Suicidal behavior	Suicide attempt Mean	Suicide attempt SD	Depressed Mean	Depressed SD	Health Mean	Health SD	Methods for Cortisol Measurement	Journal Study design	AHRQ	NOS
Zhenghua Wang, 2014 (37)	2014	China	18-60	89	62	Plasma	Morning	SI	387.63	191.88	529.58	165.6			Electrochemiluminescent immunoassay (ECLIA)	cross-sectional	7	
Haibin Zhu et al., 2016 (38)	2016	China	18-45	112	57	Serum	Morning	SI+SA	0.629	0.329	0.54	0.29			Radioimmunoassay(RIA)	cross-sectional	8	
Zhenghua Wang et al., 2017 (39)	2017	China	18-60	877	/	Plasma	Morning	SI	529.58	165.6	437.63	176.46	452.71	135.57	Electrochemiluminescent immunoassay (ECLIA)	cross-sectional	7	
Yanhua Wang et al., 2021 (40)	2021	China	18-65(45.76 ± 14.01)	158	107	Plasma	Morning	SA	20.31	6.62	19.18	6.7			Electrochemiluminescent immunoassay (ECLIA)	cross-sectional	7	
Yang Zhang, 2023 (41)	2023	China	6-18	150	74	blood	Morning	SI	324	123	359	204.4			Electrochemiluminescent immunoassay (ECLIA)	cross-sectional	7	
R. Strumila et al., 2024 (42)	2024	Lithuania	Patient(40.68 ± 16.76)/ ( 33.49 ± 12.77)/	156	108	Plasma	Morning	SA	351.08	134.54	403.96	111.77	384.19	129.04	/	case-control observational		8
B. Peng et al., 2024 (43)	2024	China	12-35	130	116	Serum	Morning	SA	242.58	130.8	293.26	106.76			Enzyme-linked immunosorbent assay (ELISA)	cross-sectional	9	
Jae-Min Kim et al., 2023 (44)	2023	Korea	17-85(56.9 ± 14.9)	1094	753	Serum	Morning	SA	0.397	0.226	0.3705	0.216			Electrochemiluminescent immunoassay (ECLIA)	cohort study		8
A. Karabatsiakis et al., 2022 (45)	2022	Germany	(55.09 ± 16.82)/(58.5 ± 6.3)/(57 ± 5)	77	49	Hair	/	SC	1.137	1.319	0.348	0.19	0.207	0.055	Enzyme-linked immunosorbent assays (ELISA)	cross-sectional	7	
A. D. Genis-Mendoza et al., 2022 (21)	2022	Mexico	18-55	112	45	Plasma	Morning	SA	404.46	423.61			242.95	185.45	Enzyme-linked immunosorbent assays (ELISA)	cross-sectional	8	
J. M. Hennings et al., 2021 (46)	2021	Germany	47.2 ± 13.4	205	162	Plasma	Morning	SI	501.8	156.7	523.7	190.8			Radioimmunoassay (RIA)	cohort study		8
M. M. Rizk et al., 2018 (47)	2018	USA	18-65 (33.6 ± 10.3)	58	34	saliva	/	SI	1.96	0.727	1.726	0.402	1.764	0.442	Radioimmunoassay (RIA)	cross-sectional	7	
C.-C. Chang et al., 2017 (48)	2017	China	20-64	119	47	Serum	Morning	SI	0.3257	0.1398	0.327	0.123			Chemiluminescence Immunoassay (CLIA)	cross-sectional	7	
W. Pitchot et al., 2005 (49)	2005	Belgium	(36.9 ± 14.9)/(43.2 ± 10.4)/(38.6 ± 11.8)	60	36	Blood	Morning	SA	2.8448	0.7728	3.174	2.07	3.2528	1.4352	Radioimmunoassay (RIA)	cross-sectional	7	
Inder, W. J et al., 1997 (50)	1997	New Zealand	(32.5 ± 1.5)/(32.5 ± 1.5)/(32.5 ± 3)	56	34	Plasma	Afternoon	SA	331	90	243	27	239	37	Enzyme-linked immunosorbent assay (ELISA)	cross-sectional	7	

SI, Suicidal Ideation; SA, Suicide Attempts; SC, Suicide Complet.

### 3.3 Meta-analysis results

This meta-analysis adhered to the Preferred Reporting Items for Systematic Reviews and Meta-Analyses (PRISMA) Checklist and guidelines. Forest plots were generated to report the standardized mean differences (SMDs) and their 95% confidence intervals (CIs), and calculations were performed for each included study. Heterogeneity among studies was evaluated using the I² statistic and Q-test. For analyses with significant heterogeneity (I² > 50% or P < 0.05), a random-effects model with the DerSimonian-Laird estimator was applied. Potential publication bias was assessed using Begg’s funnel plot and Egger’s regression test, with statistical significance set at P < 0.05. The overall stability of the results was examined using sensitivity analyses, which involved systematically excluding one study at a time from the pooled dataset to evaluate the robustness of the findings.

**Figure 2:**
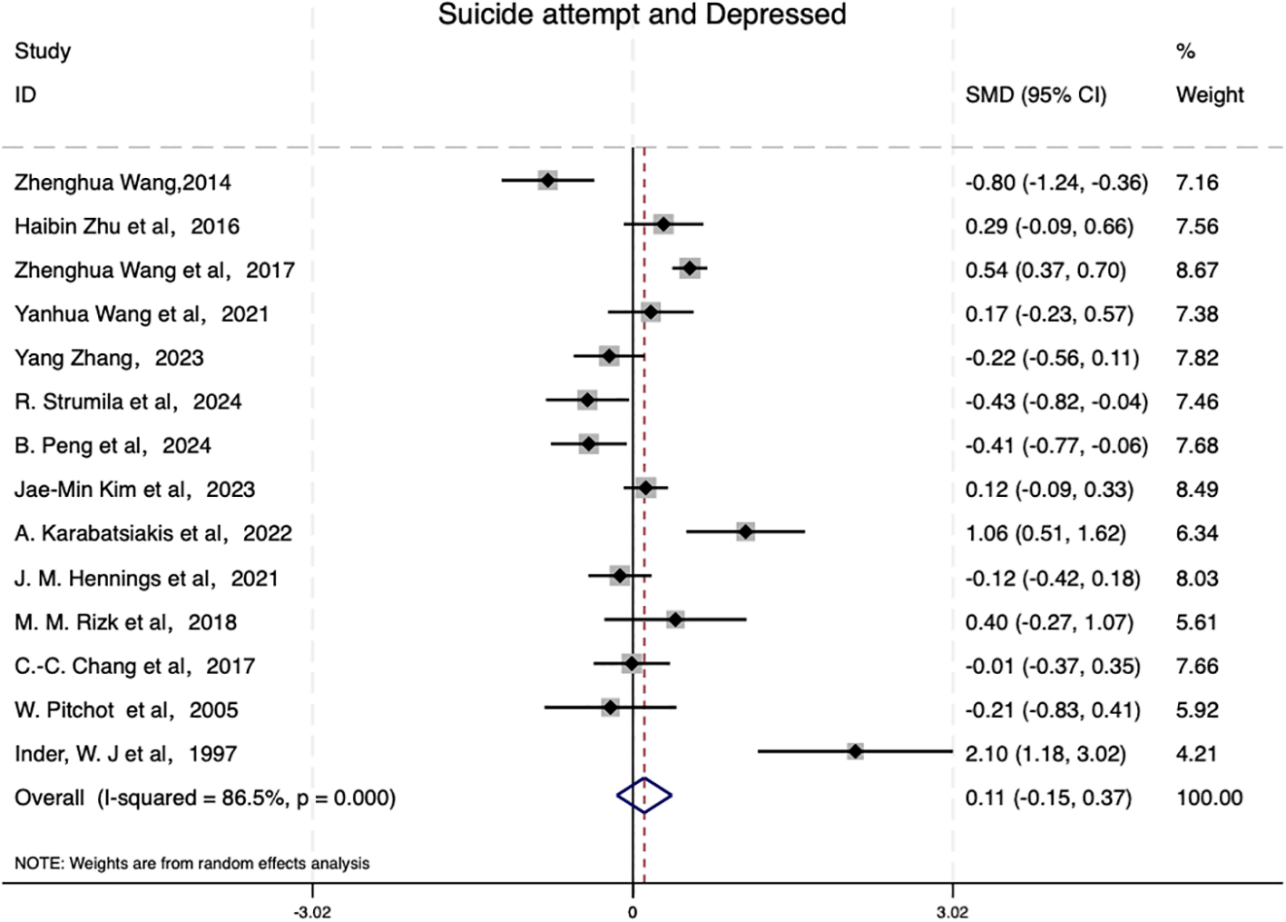
The association between cortisol levels in patients with suicide attempt and depressed.

The results of this meta-analysis are presented in **Figure 2**, showing that the overall standardized mean difference between the group with suicidal behavior and the group without suicidal behavior (SMD =0.108,95% CI: [-0.151,0.367], P = 0.413) indicating no statistically significant effect size between the two groups. Additionally, the heterogeneity test revealed significant heterogeneity among the studies (I^2^ = 86.5%, τ^2^ = 0.1944,  P <0.001), suggesting that differences across studies might substantially influence the pooled effect size. The distribution of the study weights highlighted inconsistent effect directions, and some studies showed a positive effect. Inder et al. (50) (SMD = 2.099, 95% CI [1.179, 3.019]) and Karabatsiakis et al. (45) (SMD = 1.064, 95% CI [0.505, 1.623]) reported that cortisol levels in depressed patients with suicidal behavior were significantly higher than those in patients without suicidal behavior. However, others [e.g., W. Pitchot et al. (49) and Wang (37)] have demonstrated negative effects.

**Figure 3:**
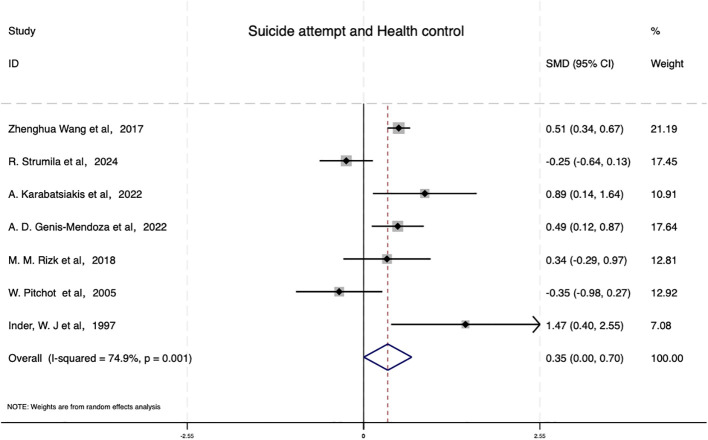
The association between cortisol levels in patients with suicide attempt and health control.

Compared to healthy controls, as shown in **Figure 3**, the pooled effect size for patients with depression and suicidal behavior was (SMD=0.350,95% CI: [0.003,0.696], P = 0.048), reaching statistical significance. This indicates a modest but significant difference in cortisol levels between the case and control groups.

**Figure 4:**
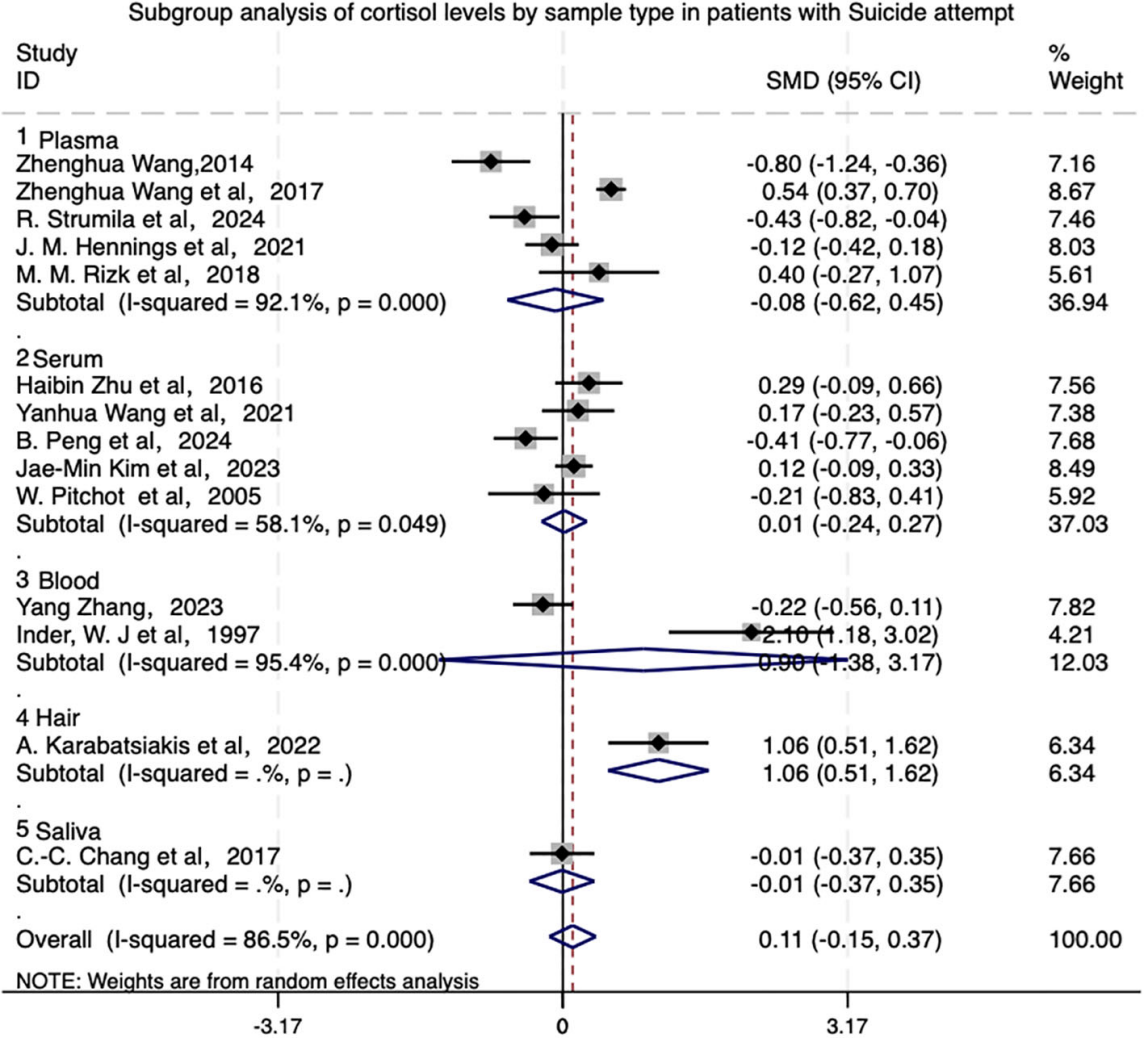
Subgroup analysis of cortisol levels by sample type (e.g., blood, plasma, saliva, hair) in patients with suicide attempt.

The high heterogeneity may be attributed to differences in the study design, sample characteristics, or interventions protocols. As shown in **Figure 4**, subgroup analyses of cortisol sample types in depressed patients demonstrated no overall statistical significance (P>0.05). However, hair sample analyses showed a significant positive effect (SMD=1.06) with stable results, potentially reflecting chronic stress levels, while other sample types (blood, plasma, whole blood) showed no significant differences and higher heterogeneity.

**Figure 5:**
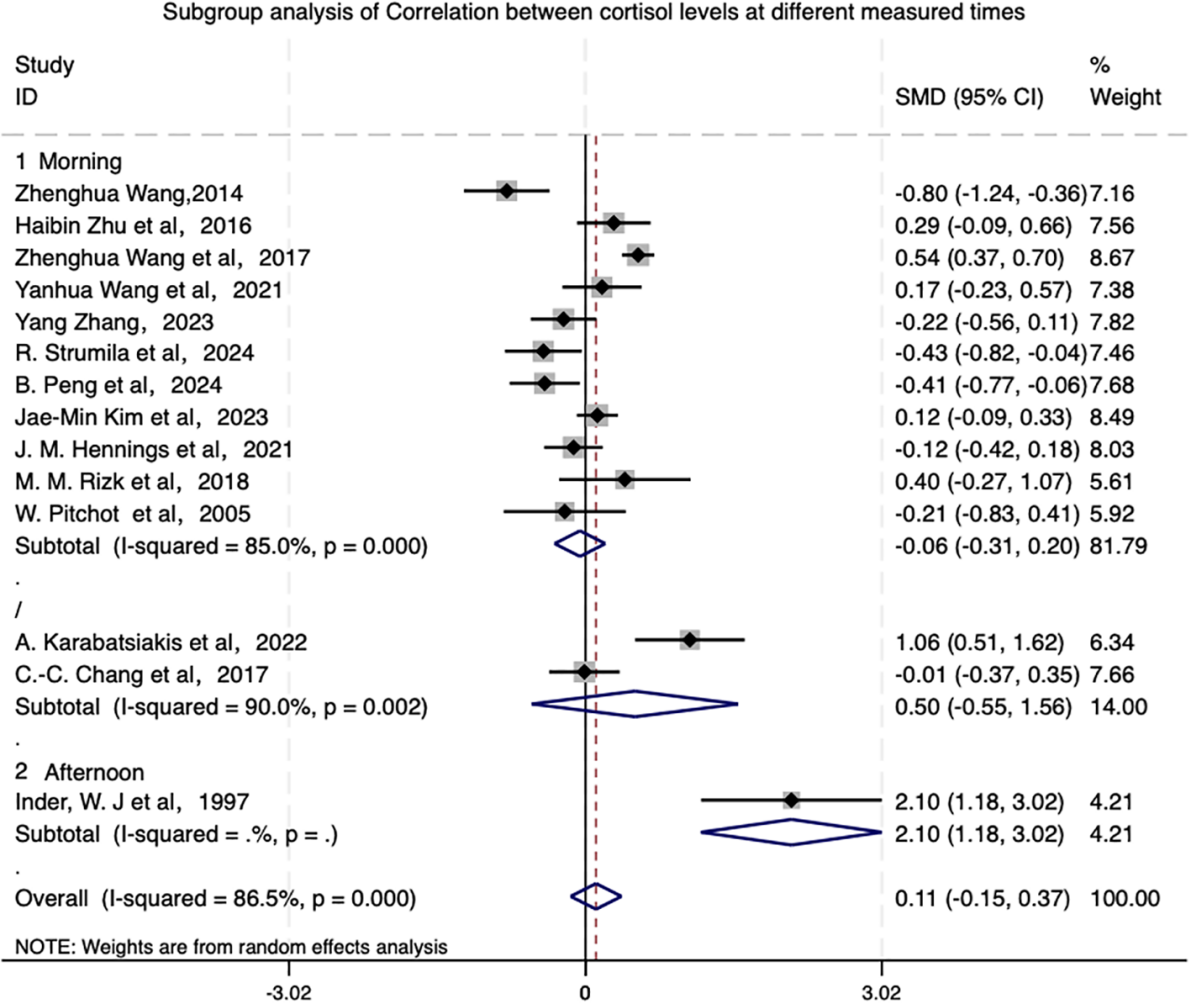
Subgroup analysis of correlation between cortisol levels at different measured times.

Subgroup analyses of measurement timing in depressed patients are presented in **Figure 5**. We found no significant association between morning cortisol levels and suicidal behavior (SMD=-0.055, 95% CI [-0.314, 0.203]), suggesting that morning cortisol levels may not be a strong predictor of suicidal behavior.

**Figure 6:**
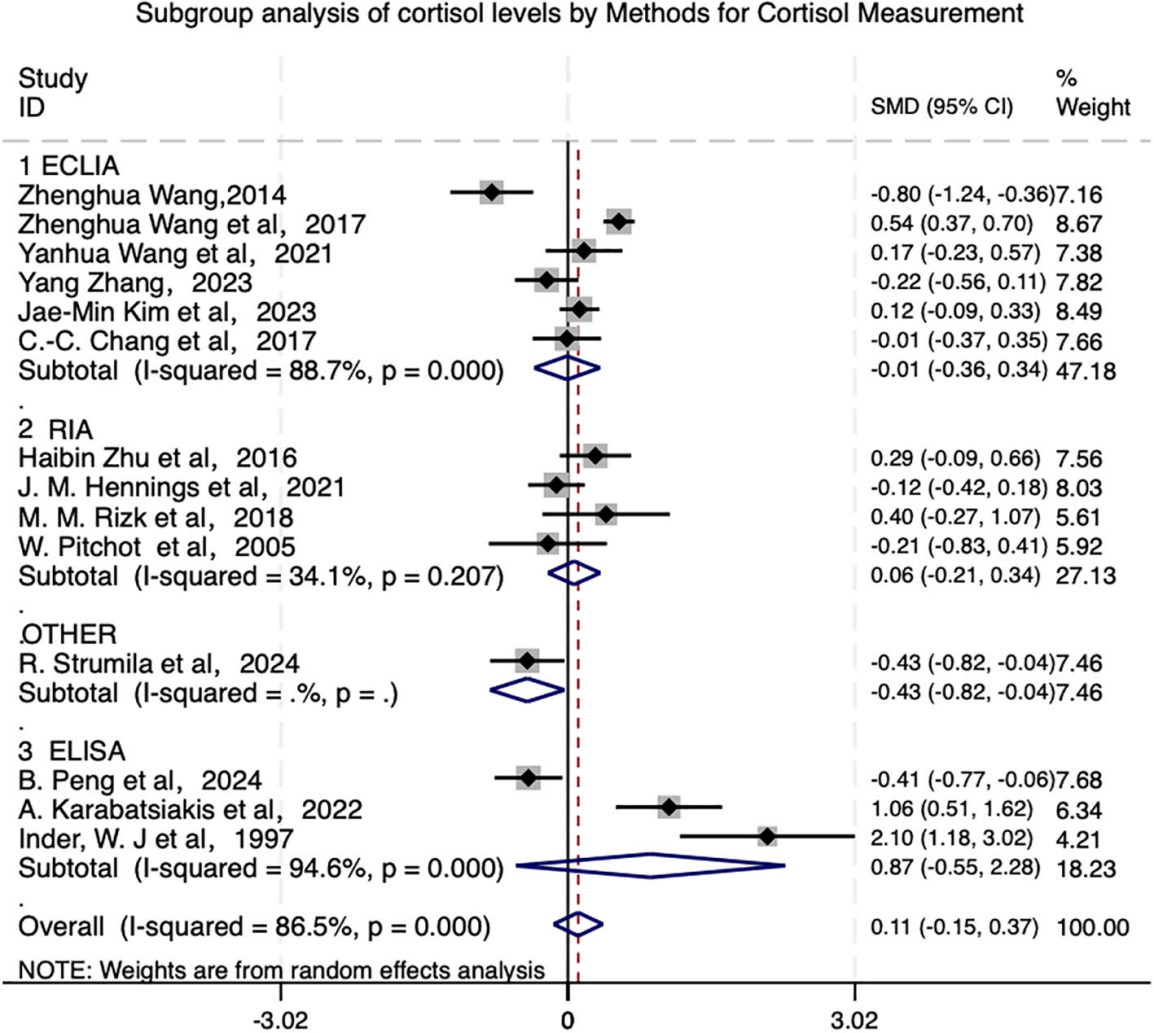
Subgroup analysis of cortisol levels by methods for cortisol measurement.

Subgroup analyses comparing three cortisol measurement methods in depressed patients — electrochemiluminescence immunoassay (ECLIA), radioimmunoassay (RIA), and enzyme-linked immunosorbent assay (ELISA) — revealed no statistically significant differences between subgroups (P>0.05) in **Figure 6**. Significance tests for each subgroup showed: ECLIA subgroup: Non-significant pooled effect size (SMD=-0.009, 95% CI [-0.355, 0.337], p=0.960), indicating no significant difference in cortisol levels between suicidal patients and controls under ECLIA methodology. RIA subgroup: SMD=0.065 (95% CI [-0.211, 0.341], p=0.645). ELISA subgroup: SMD=0.868 (95% CI [-0.547, 2.283], p=0.229), with extreme between-study effect size variations. For example, Inder et al. (50) (SMD=2.099) demonstrated a strongly positive effect, whereas B. Peng et al. (43) (SMD=-0.415) showed a negative effect. Contradictory effect directions were also observed between studies by the same research group (e.g., Wang (37) [SMD=-0.801] *vs*. Zhenghua Wang et al. (39) [SMD=0.537]). indicating some, albeit limited, influence of the variable. These findings highlight the complexity of the relationship between cortisol levels, depression, and suicidal behavior, and underscore the need for further research to disentangle the effects of biological, psychological, and methodological factors.

### 3.4 Sensitivity analysis

Sensitivity analyses were conducted for results with I^2^ ≥50%, focusing on plasma, serum, and whole blood subgroups. Following these subgroup analyses, the heterogeneity showed no significant changes **Supplementary Figure S1**. The estimated effect sizes ranged from 0.019-0.171, with wide confidence intervals spanning from negative to positive values, indicating no significant difference in the overall results, regardless of which study was excluded. When individual studies were excluded individually, the pooled effect size remained within the range of 0.019-0.171, with no statistical significance, suggesting that the overall findings were robust. The largest effect size (Hedges’ g=0.171) was observed when Wang (37) was excluded, while the smallest effect size (Hedges’ g=0.019) was observed when Inder et al. (50) was excluded. These results suggest that certain studies, such as those by Wang (37) and Inder et al. may have influenced the pooled results to some extent. Therefore, further attention should be paid to the characteristics and potential biases of these studies to better understand their impact on meta-analysis findings.

### 3.5 Publication bias

Egger's test indicated potential publication bias (P=0.048), while Begg's test showed no significant bias (P=0.661). The funnel plot demonstrated roughly symmetrical distribution around the summary effect size (SMD=0.14, 95% CI [0.052, 0.227]) in **Supplementary Figure S2**. Combined results from Egger's test, Begg's test, and the funnel plot suggested that while Begg's test detected no significant publication bias, Egger's test raised concerns about potential bias. However, the trim-and-fill method revealed no missing studies requiring imputation, further supporting the robustness of the findings.

### 3.6 Results summary

General Characteristics: As shown in **Table 2**, there were no statistically significant differences among the four groups in terms of age, sex, BMI, smoking history, or drinking history (P > 0.05). After excluding the healthy control group, no significant differences in disease duration were observed among the remaining three groups (P = 0.073). However, there were significant differences in the occurrence of stressful events within the past three months among the healthy controls, depression without suicidal ideation, depression with suicidal ideation, and depression with suicidal behavior groups (P < 0.001). **Table 2** presents the results.

**Table 2:** Comparison of general characteristics among groups.

Variable	Healthy Controls (n=32)	Depression Without Suicidal Ideation (n=32)	Depression With Suicidal Ideation (n=34)	Depression With Suicidal Behavior (n=33)	*F/χ^2^/H*	*P*
Age (years)	29.83 ± 8.35	34.00 ± 12.81	34.39 ± 15.77	28.04 ± 16.82	6.77	0.080
Gender					2.76	0.430
Male	7 (15.63%)	7 (15.63%)	5 (14.71%)	9 (27.27%)		
Female	25 (84.37%)	25 (84.37%)	29 (85.29%)	24(72.73%)		
BMI (kg/m^2^)	22.01 ± 2.32	21.37 ± 3.31	22.48 ± 3.66	21.40 ± 3.01	0.86	0.465
Disease duration (years)	——	5.00 ± 6.34	6.18 ± 8.67	3.64 ± 4.15	6.96	0.073
Smoking history					3.21	0.564
Yes	7 (15.63%)	6 (18.75%)	4 (11.76%)	3 (9.09%)		
No	25 (84.37%)	26(81.25%)	30 (88.24%)	30 (90.9%)		
Drinking history					3.41	0.781
Yes	2 (6.25%)	1 (3.13%)	2 (5.88%)	1 (3.03%)		
No	30 (93.75 %)	31 (96.87%)	32 (94.12%)	32 (96.97%)		
Stressful events in past 3 months					21.41	<0.001
Yes	0 (0.00%)	4 (12.50%)	4 (11.76%)	15(45.45%)		
No	32 (100.00%)	28 (87.50%)	30 (88.24%)	18(54.55%)		

The HAMD scores for the four groups were as follows: 0.90 ± 0.80 in the healthy control group, 23.58 ± 3.76 in the depression without suicidal ideation group, 25.21 ± 5.17 in the depression with suicidal ideation group, and 29.71 ± 5.41 in the suicidal behavior group. The differences in the HAMD scores among the groups were statistically significant (P < 0.001).

Hair Cortisol Levels (HCL): At the 1-cm segment level, the HCL were 4.47 ± 1.75 ng/mg in the healthy control group, 5.43 ± 2.42 ng/mg in the depression without suicidal ideation group, 5.04 ± 2.30 ng/mg in the depression with suicidal ideation group, and 3.46 ± 1.92 ng/mg in the depression with suicidal behavior group. Significant differences in HCL were observed among the four groups (P = 0.004). Multiple comparisons revealed that the HCL of the depression with suicidal behavior group was significantly lower than that of both the depression without suicidal ideation group (k:P = 0.003) and the depression with suicidal ideation group (l: P = 0.037). The HCL of the depression with suicidal behavior group was slightly lower than that of the healthy control group, but the difference was not statistically significant (i: P = 0.183). No significant differences were found among the healthy control group, depression without suicidal ideation group, and depression with suicidal ideation group (g, h, j: P > 0.05). **Table 3** presents the results.

**Table 3:** 0-1cm 1-2cmHCL.

Groups	n	0-1cm HCL(ng/mg)	*H*	*P*	1-2cm HCL(ng/mg)	*H*	*P*
Healthy Controls	32	4.47 ± 1.75	13.40	0.004	4.47 ± 1.69	11.98	0.007
Depression Without Suicidal Ideation	32	5.43 ± 2.42^g^			5.65 ± 3.59^m^		
Depression With Suicidal Ideation	34	5.04 ± 2.30^h, j^			5.10 ± 2.88^n, p^		
Depression With Suicidal Behavior	33	3.46 ± 1.92^i, k, l^			3.21 ± 1.47^o, q, r^		

HCL, hair cortisol levels; Compared to the Healthy Control Group, g: P=0.885, h: P=1.000, i: P=0.183; Compared to the Depression Without Suicidal Ideation Group, j: P=1.000, k: P=0.003; Compared to the Depression With Suicidal Ideation Group, l: P=0.037; Compared to the Healthy Control Group, m: P =1.000, n: P =1.000; o: P =0.077; Compared to the Depression Without Suicidal Ideation Group, p: P =1.000, q: P =0.009; Compared to the Depression With Suicidal Ideation Group, r: P =0.050.

**Figure 7:**
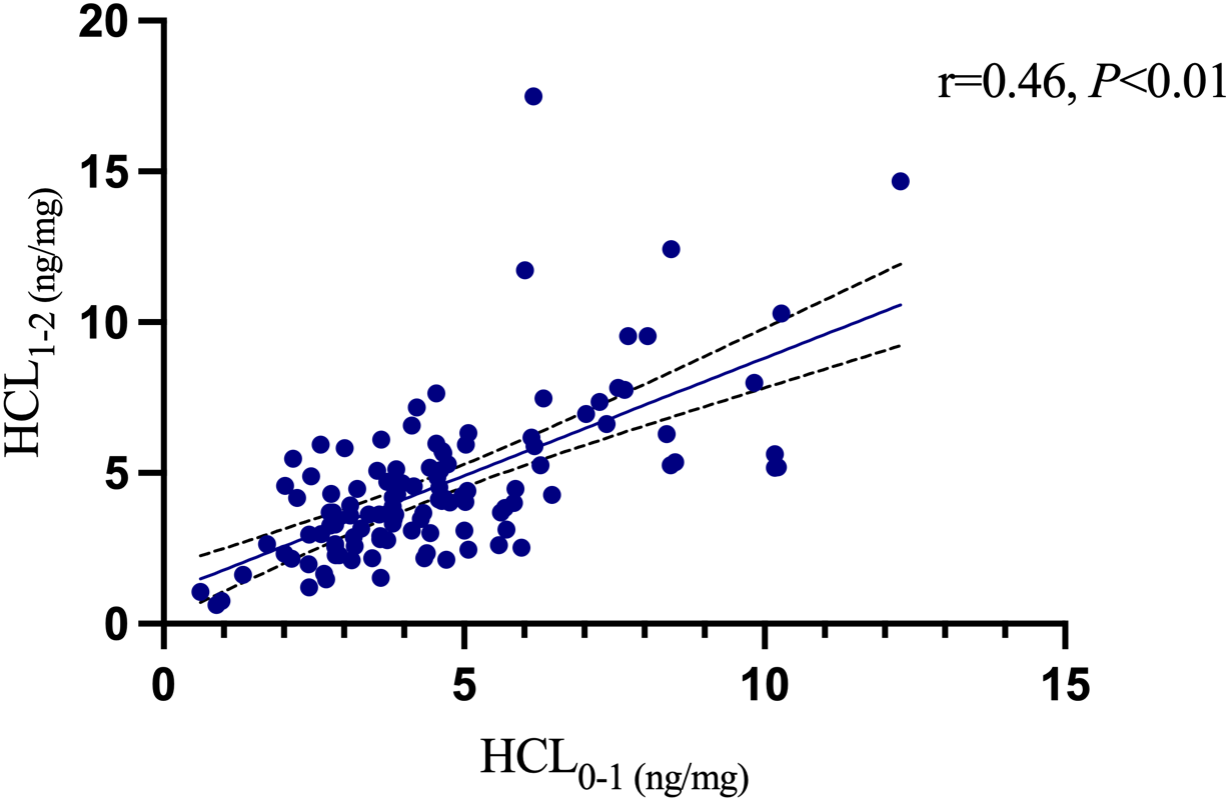
Compare 0-1cm HCL with 1-2cm HCL.

**Figure 8:**
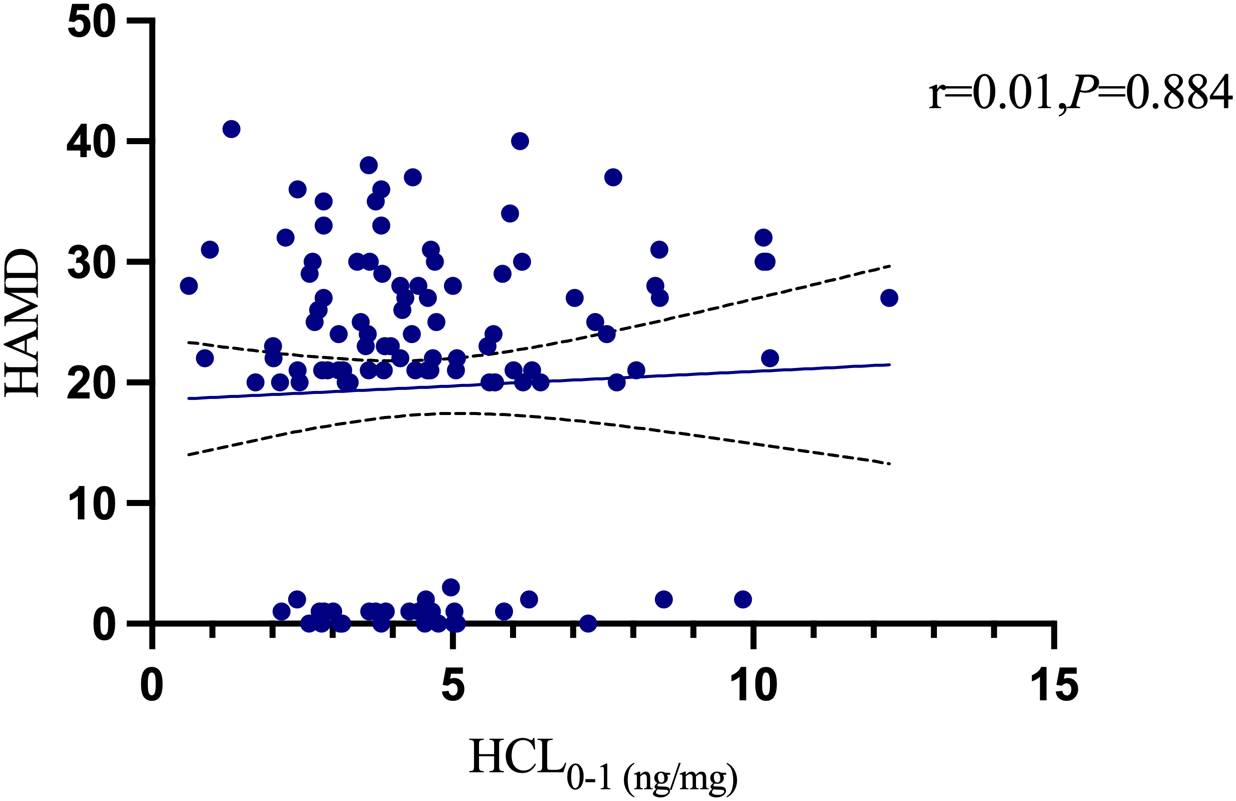
0-1cmHCL and HAMD.

**Figure 9:**
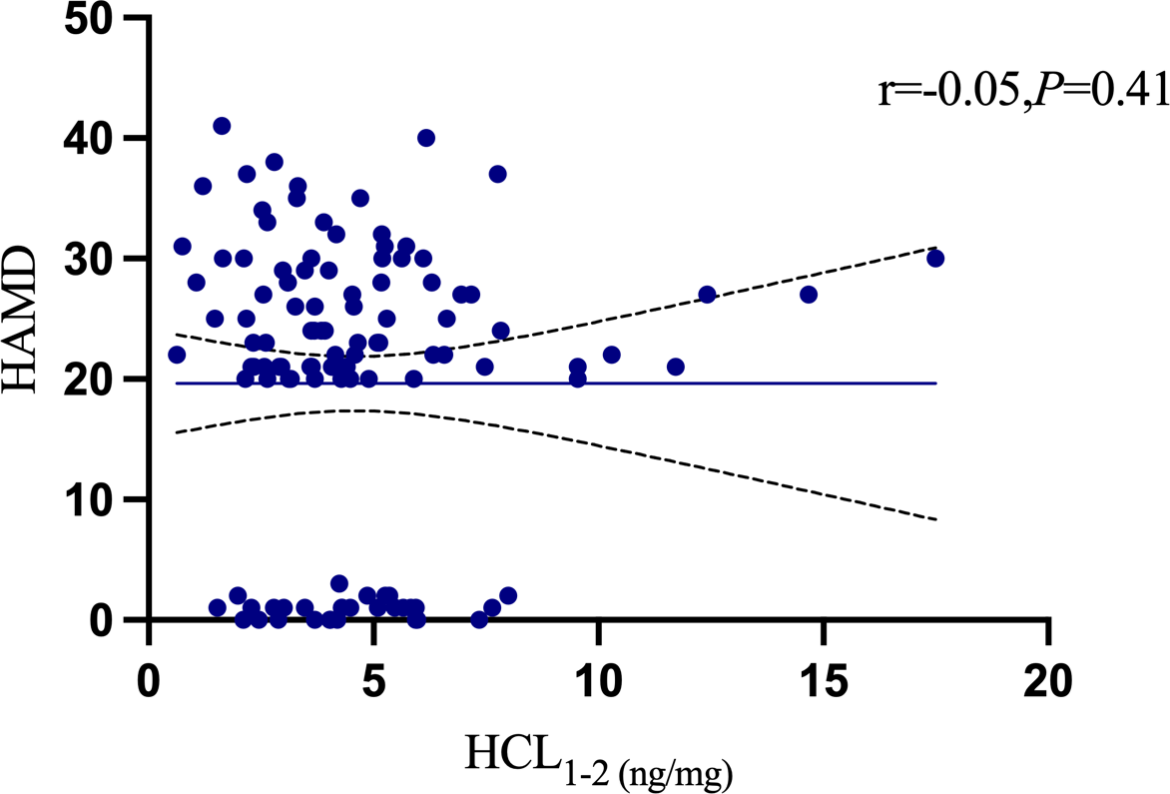
1-2cmHCL and HAMD.

At the 1–2 cm segment level, the HCL were 4.47 ± 1.69 ng/mg in the healthy control group, 5.65 ± 3.59 ng/mg in the depression without suicidal ideation group, 5.10 ± 2.88 ng/mg in the depression with suicidal ideation group, and 3.21 ± 1.47 ng/mg in the depression with suicidal behavior group. Significant differences in HCL were observed among the four groups (P = 0.007). Multiple comparisons revealed that the HCL of the depression with suicidal behavior group was significantly lower than that of both the depression without suicidal ideation group (q: P = 0.009) and depression with suicidal ideation group (r: P = 0.050). The HCL of the depression with suicidal behavior group was slightly lower than that of the healthy control group, but the difference was not statistically significant (o: P = 0.077). No significant differences were found among the healthy control group, depression without suicidal ideation group, and depression with suicidal ideation group (m, n, p: P > 0.05). **Table 4** presents the results. There were no significant differences in HCL between the 0–1 cm and 1–2 cm segments among the four groups (P > 0.05). Pearson correlation analysis of the 131 participants showed a positive correlation between the 0–1 cm and 1–2 cm HCL levels (r = 0.46, P < 0.001), as shown in **Figure 7**. Further analysis using Spearman’s correlation showed no significant association between 0–1 cm or 1–2 cm HCL and HAMD scores (P = 0.884 and P = 0.410, respectively), as shown in **Figures 8**, **9**.

**Table 4:** Under the curve (AUC), sensitivity, specificity, and cut-off values.

	AUC (95%)	Sensitivity (%)	Sensitivity (%)	Cut-off
HCL0-1	0.728 (0.614, 0.842)	89.3%	52.6%	4.72
HCL1-2	0.713 (0.602, 0.824)	89.3%	54.4%	4.80

Association Between Suicidal Behavior and HCL: To further analyze the association between suicidal behavior and hair cortisol levels, participants with depression and suicidal behavior (n = 32, 24.43%) were defined as the suicidal behavior group, while the remaining three groups (n = 99, 75.57%) were defined as the non-suicidal behavior group. The 0–1 cm HCL in the suicidal behavior group was 3.46 ± 1.92 ng/mg, and the 1–2 cm HCL was 3.21 ± 1.47 ng/mg. In the non-suicidal behavior group, the 0–1 cm HCL was 4.95 ± 2.17 ng/mg, and the 1–2 cm HCL was 5.03 ± 2.78 ng/mg. Spearman correlation analysis showed significant negative correlations between suicidal behavior and the 0–1 cm HCL (r = -0.314, P = 0.001) and 1–2 cm HCL (r = -0.312, P = 0.001).

Predictive Value of HCL for Suicidal Behavior: The predictive value of hair cortisol levels for suicidal behavior was evaluated using receiver operating characteristic (ROC) curves and the area under the curve (AUC). The AUC for 0–1 cm HCL was 0.728 (95% CI: 0.614-0.842), with an optimal cut-off value of 4.72 ng/mg, sensitivity of 89.3%, and specificity of 52.6%. The AUC for 1–2 cm HCL was 0.713 (95% CI: 0.602-0.824), with an optimal cutoff value of 4.80 ng/mg, a sensitivity of 89.3%, and a specificity of 54.4%.

## 4 Discussion

Activation of the hypothalamic-pituitary-adrenal (HPA) axis occurs in response to physiological and psychological stressors. Initial stress triggers excessive activation of the HPA axis, leading to elevated cortisol levels (51). Cortisol regulates physiological responses by binding to glucocorticoid receptors (GR) and mineralocorticoid receptors (MR). At this stage, GR-mediated negative feedback mechanisms remain functional in maintaining homeostasis (51), where cortisol binding to GR/MR suppresses ACTH secretion and reduces cortisol release (52). Under chronic stress, GR dysfunction — characterized by downregulated expression or reduced sensitivity — weakens negative feedback regulation, resulting in HPA axis hyperactivation followed by compensatory exhaustion, reduced basal cortisol level (53), and blunted stress responses (54). Current evidence suggests that HPA axis activity in depressed patients may manifest as either chronic hyperactivity (e.g., sustained cortisol hypersecretion under prolonged stress) or functional exhaustion (e.g., cortisol suppression in individuals with repeated suicide attempts). These divergent patterns may explain the nonlinear relationship between cortisol levels and suicidal behavior: while some studies associate suicidality with hypercortisolemia (21), others report strong correlations with hypocortisolemia (55).

Previous research on cortisol and suicidal behavior has largely focused on the dexamethasone suppression test (DST). Dexamethasone, an exogenous glucocorticoid, binds to GRs in the hypothalamus and pituitary, triggering hypothalamic-pituitary-adrenal (HPA) axis feedback and reducing cortisol secretion. In individuals with glucocorticoid resistance, cortisol levels remain unaltered despite feedback, indicating hypothalamic-pituitary-adrenal (HPA) axis hyperactivity. Although DST was among the first biological markers proposed in psychiatry, but its findings have not been widely replicated, and the test has been criticized for its inability to accurately simulate endogenous HPA responses to naturally occurring stressors (56). Depressed patients may exhibit HPA axis abnormalities as either hyperactivity or hypoactivity, a heterogeneity that DST fails to adequately capture (57). Although DST demonstrates 95% specificity in melancholic depression, its sensitivity is only 67% (56), implying potential underdiagnosis in approximately one-third of patients. In suicide prediction, DST non-suppression status is associated with suicide risk, yet its positive predictive value remains limited (58). Additionally, obese or metabolically compromised patients may exhibit accelerated dexamethasone metabolism, leading to false-positive results (59).Recent studies have demonstrated that the combined dexamethasone-CRH test (DEX-CRH test) — sequentially administering dexamethasone (to suppress endogenous CRH) and exogenous CRH — can more sensitively detect HPA axis negative feedback deficits and central CRH hypersensitivity (57, 60). One study found that repeated suicide attempters persistently exhibit blunted HPA axis reactivity, indicating sustained HPA axis dysfunction (61). Furthermore, the cortisol awakening response (CAR), assessed through post-awakening cortisol dynamics, reflects basal HPA axis functionality. Depressed patients with suicidal ideation frequently demonstrate elevated CAR magnitude, an abnormality that remains significant even after adjusting for prior depressive symptoms (62).

This study found that depressed patients with suicidal behavior exhibited significantly higher cortisol levels compared to healthy controls (SMD=0.350, 95% CI [0.003, 0.696]), whereas no significant difference was observed relative to depressed patients without suicidal behavior (SMD=0.108, 95% CI [-0.125, 0.341]). These findings may reflect a complex interplay between general dysregulation of the hypothalamic-pituitary-adrenal (HPA) axis and suicidality-specific physiological states. General dysregulation of the HPA axis may represent a common feature of depression. In heterogeneity and subgroup analyses, differences in sample types were observed: hair samples (SMD=1.06) showed distinct effect sizes compared to plasma, serum, and whole blood. Hair cortisol reflects cumulative exposure over the preceding 1–2 months (63), whereas blood samples capture instantaneous concentrations at the time of collection. Given cortisol’s short half-life (60–90 minutes) (64), blood-based measurements are more susceptible to circadian rhythm influences (e.g., morning peaks) and acute stress interference (64).

Cortisol's diurnal rhythm typically exhibits a morning peak — the cortisol awakening response (CAR) — reaching maximum levels 30–60 minutes post-awakening, followed by a gradual decline to nadir concentrations at night (65, 66). CAR is recognized as a key indicator of HPA axis activity, with its magnitude and temporal profile potentially modulated by sleep quality, awakening time, and chronotype. For instance, higher sleep efficiency and longer total sleep duration correlate with enhanced CAR (67), whereas night shift work may induce misalignment between cortisol peaks and sleep-wake cycles (68). In subgroup analyses of sampling timeframes, morning measurements were defined as 6:00–11:00 AM across studies (primarily 6:00–8:30 AM). However, the lack of standardized sampling schedules likely contributed to inconsistent capture of CAR peaks between studies.

In another subgroup analysis examining methodological differences, variations in sensitivity and specificity across detection techniques (ECLIA, RIA, ELISA) contributed to inconsistent effect sizes. The high heterogeneity observed in the ELISA subgroup may be attributable to non-standardized antibodies or calibrators (69). ECLIA demonstrates higher specificity for free cortisol detection with minimal cross-reactivity (e.g., no corticosterone interference) (70). Although RIA and ECLIA exhibit superior stability, insufficient sample sizes or clinical context variability may account for their non-significant effect sizes. Current evidence indicates that methodological choices — particularly the lack of standardization in ELISA — constitute a major source of heterogeneity, necessitating unified protocols through multicenter collaboration. Despite the biological plausibility of cortisol-suicidality associations, no single detection method reliably captures this relationship. Future studies should integrate methodological optimization with clinically stratified designs, such as combining multiple techniques (e.g., ECLIA+RIA) to validate results (71).

This study analyzed the HAMD scores and hair cortisol levels (HCL) of all participants and found that the hair cortisol levels in the suicidal behavior group were significantly lower than those in the other three groups. There was no significant difference between the 0–1 cm HCL and 1–2 cm HCL within each group, and no correlation was observed between the HAMD scores and HCL. However, a negative correlation was identified between the 0–1 cm HCL and 1–2 cm HCL and suicidal behavior (r = -0.314; r = -0.312), suggesting that higher hair cortisol levels are associated with a lower likelihood of suicidal behavior.

Further analysis of the negative correlation between HCL and suicidal behavior led to the construction of receiver operating characteristic (ROC) curves. As shown in **Table 4**. The results showed that the area under the curve (AUC) for 0–1 cm HCL was 0.728, with an optimal cut-off value of 4.72 ng/mg, and the AUC for 1–2 cm HCL was 0.713, with an optimal cut-off value of 4.80 ng/mg. The predictive sensitivity was 89.3%, demonstrating that hair cortisol levels have a certain degree of accuracy in predicting suicidal behavior. This is a novel finding of this study, as there are currently few similar studies conducted in clinical settings. Future research is needed to provide more evidence and validate hair cortisol as a specific molecular marker for predicting suicidal behavior.

These findings suggest that hair cortisol concentration has limited screening value as a standalone biomarker but may serve as a component in multimodal predictive models. For instance, combining cortisol with biomarkers such as cholesterol and folate can improve prediction accuracy for fatal/non-fatal suicide attempts (44). Hair cortisol concentration reflects long-term (weeks to months) HPA axis activity and provides an objective measure of chronic stress (72). Chronic stress-induced HPA axis dysregulation is strongly associated with suicidal behavior. For example, studies demonstrate significantly elevated peripheral plasma cortisol levels in individuals with multiple suicide attempts (21), whereas longitudinal monitoring via hair cortisol concentration may more reliably capture such abnormalities (73). Several studies have identified elevated hair cortisol concentration as a risk factor for suicidal behaviors (SBs), particularly among depressed adolescents and recurrent suicide attempters (72). One study specifically reported a significant association between hair cortisol concentration levels and suicide attempt history in depressed adolescents (74). In summary, although the application of hair cortisol levels (HCL) as a standalone measure is limited, its advantages in chronic stress assessment can complement existing clinical tools. Future research should investigate the synergistic effects of HCL with other biomarkers, such as inflammatory markers and epigenetic indicators, as well as its integration with Delphi-consensus-recommended standardized clinical pathways, including dynamic risk assessment and multidisciplinary team collaboration (75). This approach may enhance the precision of suicide risk prediction and its translational value in clinical practice.

Limitations: 1) The most critical limitation is the significant heterogeneity across included studies, where variations in cortisol sampling (sample types), collection timing, and detection methodologies may have influenced the results. 2) The inclusion of both Chinese- and English-language literature with multinational populations may introduce language bias and baseline disparities (e.g., differences in illness duration, comorbidities). 3) Geographical diversity in study populations, inconsistent depression assessment tools, and heterogeneous intervention protocols further confound the findings. 4) The limited number of studies precluded subgroup analyses by sex, socioeconomic status, or measurement units. 5) Most included studies were cross-sectional; future longitudinal investigations should track cortisol dynamics across phases (baseline, crisis, remission) to clarify associations with suicidal depression. 6) For hair cortisol analysis, establishing standardized detection protocols and expanding sample sizes are needed to validate the reliability of hair cortisol concentration.

## 5 Results

The meta-analysis indicated that cortisol levels in depressed patients with suicidal behavior were significantly higher than those in healthy individuals (SMD = 0.350, 95% CI [0.003, 0.696]). However, the cortisol levels in patients with suicidal behavior were only slightly higher than those in patients without suicidal behavior, and the pooled effect size (SMD = 0.108, 95% CI [-0.151, 0.367]) did not show a significant difference. This finding may reflect complexity of overall HPA axis dysregulation in depression and the specific physiological states influencing cortisol levels in patients with suicidal behavior. The experiment revealed that patients with depression and suicidal behavior had lower hair cortisol levels (HCL) than those with depression without suicidal behavior.

## Data Availability

The datasets for this study are available from the corresponding author upon reasonable request.
