# Supplementary material for: Cortisol levels and depression suicide risk: a combined exploration of meta-analysis and case-control study
Source: Front Psychiatry. 2025 Apr 30;16:1563819. doi: 10.3389/fpsyt.2025.1563819 (PMC12076087; doi:10.3389/fpsyt.2025.1563819)
Supplement: Supplementary file 1 [file DataSheet1.docx]

Supplementary Material

# Supplementary Data

**Full search strategy for each database**

**PubMed n=269**

#1 Search:"Suicide"[Mesh]

#2 Search: (((((Suicide[Title/Abstract]) OR (Suicidal Ideation[Title/Abstract])) OR (Suicide Prevention[Title/Abstract])) OR (Suicide, Assisted[Title/Abstract])) OR (Suicide, Attempted[Title/Abstract])) OR (Suicide, Completed[Title/Abstract])

#3 #1 or #2

#4 Search: (((((((((((("Depression"[Mesh]) OR (Depression[Title/Abstract])) OR (Depressive Symptoms[Title/Abstract])) OR (Depressive Symptom[Title/Abstract])) OR (Symptom, Depressive[Title/Abstract])) OR (Emotional Depression[Title/Abstract])) OR (Depression, Emotional[Title/Abstract])) OR ((((((((((((((((((((((((((("Depressive Disorder"[Mesh]) OR (Depressive Disorder[Title/Abstract])) OR (Depressive Disorders[Title/Abstract])) OR (Disorder, Depressive[Title/Abstract])) OR (Disorders, Depressive[Title/Abstract])) OR (Neurosis, Depressive[Title/Abstract])) OR (Depressive Neuroses[Title/Abstract])) OR (Depressive Neurosis[Title/Abstract])) OR (Neuroses, Depressive[Title/Abstract])) OR (Depression, Endogenous[Title/Abstract])) OR (Depressions, Endogenous[Title/Abstract])) OR (Endogenous Depression[Title/Abstract])) OR (Endogenous Depressions[Title/Abstract])) OR (Melancholia[Title/Abstract])) OR (Melancholias[Title/Abstract])) OR (Unipolar Depression[Title/Abstract])) OR (Depression, Unipolar[Title/Abstract])) OR (Depressions, Unipolar[Title/Abstract])) OR (Unipolar Depressions[Title/Abstract])) OR (Depressive Syndrome[Title/Abstract])) OR (Depressive Syndromes[Title/Abstract])) OR (Syndrome, Depressive[Title/Abstract])) OR (Syndromes, Depressive[Title/Abstract])) OR (Depression, Neurotic[Title/Abstract])) OR (Depressions, Neurotic[Title/Abstract])) OR (Neurotic Depression[Title/Abstract])) OR (Neurotic Depressions[Title/Abstract]))) OR ((((((((((((((((((((("Depression, Postpartum"[Mesh]) OR (Depression, Postpartum[Title/Abstract])) OR (Postpartum Depression[Title/Abstract])) OR (Post-Natal Depression[Title/Abstract])) OR (Depression, Post-Natal[Title/Abstract])) OR (Post Natal Depression[Title/Abstract])) OR (Post-Partum Depression[Title/Abstract])) OR (Depression, Post-Partum[Title/Abstract])) OR (Post Partum Depression[Title/Abstract])) OR (Postnatal Depression[Title/Abstract])) OR (Depression, Postnatal[Title/Abstract])) OR (Postnatal Dysphoria[Title/Abstract])) OR (Dysphoria, Postnatal[Title/Abstract])) OR (Postpartum Dysphoria[Title/Abstract])) OR (Dysphoria, Postpartum[Title/Abstract])) OR (Post-Partum Dysphoria[Title/Abstract])) OR (Dysphoria, Post-Partum[Title/Abstract])) OR (Post Partum Dysphoria[Title/Abstract])) OR (Post-Natal Dysphoria[Title/Abstract])) OR (Dysphoria, Post-Natal[Title/Abstract])) OR (Post Natal Dysphoria[Title/Abstract]))) OR ((((((((((((((((((((((("Depressive Disorder, Treatment-Resistant"[Mesh]) OR (Depressive Disorder, Treatment-Resistant[Title/Abstract])) OR (Depressive Disorders, Treatment-Resistant[Title/Abstract])) OR (Depressive Disorder, Treatment Resistant[Title/Abstract])) OR (Disorders, Treatment-Resistant Depressive[Title/Abstract])) OR (Disorder, Treatment-Resistant Depressive[Title/Abstract])) OR (Treatment-Resistant Depressive Disorder[Title/Abstract])) OR (Treatment-Resistant Depressive Disorders[Title/Abstract])) OR (Treatment Resistant Depression[Title/Abstract])) OR (Depressions, Treatment Resistant[Title/Abstract])) OR (Depression, Treatment Resistant[Title/Abstract])) OR (Resistant Depressions, Treatment[Title/Abstract])) OR (Resistant Depression, Treatment[Title/Abstract])) OR (Treatment Resistant Depressions[Title/Abstract])) OR (Refractory Depression[Title/Abstract])) OR (Depression, Refractory[Title/Abstract])) OR (Depressions, Refractory[Title/Abstract])) OR (Refractory Depressions[Title/Abstract])) OR (Therapy-Resistant Depression[Title/Abstract])) OR (Depressions, Therapy-Resistant[Title/Abstract])) OR (Depression, Therapy-Resistant[Title/Abstract])) OR (Therapy Resistant Depression[Title/Abstract])) OR (Therapy-Resistant Depressions[Title/Abstract]))) OR ((((((((((((((((("Depressive Disorder, Major"[Mesh]) OR (Depressive Disorder, Major[Title/Abstract])) OR (Depressive Disorders, Major[Title/Abstract])) OR (Major Depressive Disorders[Title/Abstract])) OR (Major Depressive Disorder[Title/Abstract])) OR (Depression, Involutional[Title/Abstract])) OR (Involutional Depression[Title/Abstract])) OR (Melancholia, Involutional[Title/Abstract])) OR (Involutional Melancholia[Title/Abstract])) OR (Psychosis, Involutional[Title/Abstract])) OR (Involutional Psychoses[Title/Abstract])) OR (Involutional Psychosis[Title/Abstract])) OR (Psychoses, Involutional[Title/Abstract])) OR (Paraphrenia, Involutional[Title/Abstract])) OR (Involutional Paraphrenia[Title/Abstract])) OR (Involutional Paraphrenias[Title/Abstract])) OR (Paraphrenias, Involutional[Title/Abstract]))) OR ((((("Vascular Depression"[Mesh]) OR (Vascular Depression[Title/Abstract])) OR (Depression, Vascular[Title/Abstract])) OR (Subcortical Ischemic Depression[Title/Abstract])) OR (Depression, Subcortical Ischemic[Title/Abstract])))

#5 Search: (((((((((((("Hydrocortisone"[Mesh]) OR (Hydrocortisone[Title/Abstract])) OR (Pregn-4-ene-3,20-dione, 11,17,21-trihydroxy-, (11beta)-[Title/Abstract])) OR (Cortisol[Title/Abstract])) OR (Cortifair[Title/Abstract])) OR (Cortril[Title/Abstract])) OR (Hydrocortisone, (9 beta,10 alpha,11 alpha)-Isomer[Title/Abstract])) OR (Hydrocortisone, (11 alpha)-Isomer[Title/Abstract])) OR (Epicortisol[Title/Abstract])) OR (11-Epicortisol[Title/Abstract])) OR (11 Epicortisol[Title/Abstract])) OR (Cortef[Title/Abstract]))

#6 #3 AND #4 AND #5[Publication year from 1800 to 2024/09/08]

**Cochrane n=68**

#1 MeSH descriptor: [Depression] explode all trees 18552

#2 (Depression OR Depressive Symptoms OR Depressive Symptom OR Symptom, Depressive OR Emotional Depression OR Depression, Emotional):ti,ab,kw (Word variations have been searched) 119885

#3 MeSH descriptor: [Depressive Disorder] explode all trees 16766

#4 (Depressive Disorder OR Depressive Disorders OR Disorder, Depressive OR Disorders, Depressive OR Neurosis, Depressive OR Depressive Neuroses OR Depressive Neurosis OR Neuroses, Depressive OR Depression, Endogenous OR Depressions, Endogenous OR Endogenous Depression OR Endogenous Depressions OR Melancholia OR Melancholias OR Unipolar Depression OR Depression, Unipolar OR Depressions, Unipolar OR Unipolar Depressions OR Depressive Syndrome OR Depressive Syndromes OR Syndrome, Depressive OR Syndromes, Depressive OR Depression, Neurotic OR Depressions, Neurotic OR Neurotic Depression OR Neurotic Depressions):ti,ab,kw (Word variations have been searched) 59800

#5 MeSH descriptor: [Depression, Postpartum] explode all trees 1024

#6 (Depression, Postpartum OR Postpartum Depression OR Post-Natal Depression OR Depression, Post-Natal OR Post Natal Depression OR Post-Partum Depression OR Depression, Post-Partum OR Post Partum Depression OR Postnatal Depression OR Depression, Postnatal OR Postnatal Dysphoria OR Dysphoria, Postnatal OR Postpartum Dysphoria OR Dysphoria, Postpartum OR Post-Partum Dysphoria OR Dysphoria, Post-Partum OR Post Partum Dysphoria OR Post-Natal Dysphoria OR Dysphoria, Post-Natal OR Post Natal Dysphoria):ti,ab,kw (Word variations have been searched) 3893

#7 MeSH descriptor: [Depressive Disorder, Treatment-Resistant] explode all trees 772

#8 (Depressive Disorder, Treatment-Resistant OR Depressive Disorders, Treatment-Resistant OR Depressive Disorder, Treatment Resistant OR Disorders, Treatment-Resistant Depressive OR Disorder, Treatment-Resistant Depressive OR Treatment-Resistant Depressive Disorder OR Treatment-Resistant Depressive Disorders OR Treatment Resistant Depression OR Depressions, Treatment Resistant OR Depression, Treatment Resistant OR Resistant Depressions, Treatment OR Resistant Depression, Treatment OR Treatment Resistant Depressions OR Refractory Depression OR Depression, Refractory OR Depressions, Refractory OR Refractory Depressions OR Therapy-Resistant Depression OR Depressions, Therapy-Resistant OR Depression, Therapy-Resistant OR Therapy Resistant Depression OR Therapy-Resistant Depressions):ti,ab,kw (Word variations have been searched) 5730

#9 MeSH descriptor: [Depressive Disorder, Major] explode all trees 7261

#10 (Depressive Disorder, Major OR Depressive Disorders, Major OR Major Depressive Disorders OR Major Depressive Disorder OR Depression, Involutional OR Involutional Depression OR Melancholia, Involutional OR Involutional Melancholia OR Psychosis, Involutional OR Involutional Psychoses OR Involutional Psychosis OR Psychoses, Involutional OR Paraphrenia, Involutional OR Involutional Paraphrenia OR Involutional Paraphrenias OR Paraphrenias, Involutional):ti,ab,kw (Word variations have been searched) 25195

#11 MeSH descriptor: [Vascular Depression] explode all trees 2

#12 (Vascular Depression OR Depression, Vascular OR Subcortical Ischemic Depression OR Depression, Subcortical Ischemic):ti,ab,kw (Word variations have been searched) 1618

#13 #1 or #2 or #3 or #4 or #5 or #6 or #7 or #8 or #9 or #10 or #11 or #12 119996

#14 MeSH descriptor: [Hydrocortisone] explode all trees 7739

#15 (Hydrocortisone or Pregn 4 ene 3,20 dione, 11,17,21 trihydroxy , (11beta) or Cortisol or Cortifair or Cortril or Hydrocortisone, (9 beta,10 alpha,11 alpha) Isomer or Hydrocortisone, (11 alpha) Isomer or Epicortisol or 11 Epicortisol or 11 Epicortisol or Cortef):ti,ab,kw (Word variations have been searched) 17965

#16 #14 or #15 18052

#17 MeSH descriptor: [Suicide] explode all trees 2198

#18 (Suicide OR Suicidal Ideation OR Suicide Prevention OR Suicide, Assisted OR Suicide, Attempted OR Suicide, Completed):ti,ab,kw (Word variations have been searched) 9001

#19 #17 or #18 9001

#20 #13 and #16 and #19 68 [Publication year from 1800 to 2024/09/04]

**Web of science n=373**

#1 TS= (Depression) OR AB=(Depression OR Depressive Symptoms OR Depressive Symptom OR Emotional Depression)

#2 TS= (Depressive Disorder) OR AB=(Depressive Disorder OR Depressive Disorders OR Depressive Neuroses OR Depressive Neurosis OR Endogenous Depression OR Endogenous Depressions OR Melancholia OR Melancholias OR Unipolar Depression OR Unipolar Depressions OR Depressive Syndrome OR Depressive Syndromes OR Neurotic Depression OR Neurotic Depressions)

#3 TS= (Depression, Postpartum) OR AB=(Postpartum Depression OR Post-Natal Depression OR Post Natal Depression OR Post-Partum Depression OR Post Partum Depression OR Postnatal Depression OR Postnatal Dysphoria OR Postpartum Dysphoria OR Post-Partum Dysphoria OR Post Partum Dysphoria OR Post-Natal Dysphoria OR Post Natal Dysphoria)

#4 TS= (Depressive Disorder, Treatment-Resistant) OR AB=(Depressive Disorder, Treatment-Resistant OR Depressive Disorders, Treatment-Resistant OR Treatment-Resistant Depressive Disorder OR Treatment-Resistant Depressive Disorders OR Treatment Resistant Depression OR Treatment Resistant Depressions OR Refractory Depression OR Refractory Depressions OR Therapy-Resistant Depression OR Therapy Resistant Depression OR Therapy-Resistant Depressions)

#5 TS= (Depressive Disorder, Major) OR AB=(Major Depressive Disorders OR Major Depressive Disorder OR Involutional Depression OR Involutional Melancholia OR Involutional Psychoses OR Involutional Psychosis OR Involutional Paraphrenia OR Involutional Paraphrenias OR)

#6 TS= (Vascular Depression) OR AB=(Vascular Depression OR Subcortical Ischemic Depression)

#7 #1OR #2 OR #3 OR #4 OR #5 OR #6

#8 TS=(Hydrocortisone) OR AB=(Cortisol OR Cortifair OR Cortril OR Epicortisol OR 11-Epicortisol OR 11 Epicortisol OR Cortef)

#9 TS=(Suicide) OR AB=(Suicide OR Suicidal Ideation OR Suicide Prevention OR Suicide, Assisted OR Suicide, Attempted OR Suicide, Completed)

#10 #7 AND #8 AND #9 [Publication year from 1966 to 2024/09/08]

**Embase n=334**

#1 'depression'/exp 697872

#2 'central depression':ab,ti OR 'clinical depression':ab,ti OR 'depressive disease':ab,ti OR 'depressive disorder':ab,ti OR 'depressive episode':ab,ti OR 'depressive illness':ab,ti OR 'depressive personality disorder':ab,ti OR 'depressive state':ab,ti OR 'depressive symptom':ab,ti OR 'depressive syndrome':ab,ti OR 'depressivity':ab,ti OR 'mental depression':ab,ti OR 'parental depression':ab,ti OR 'depression':ab,ti 643460

#3 'hydrocortisone'/exp 164908

#4 ((('11, 17 dihydroxy 17':ab,ti AND '2 hydroxyacetyl':ab,ti AND '10, 13 dimethyl 1, 2, 6, 7, 8, 9, 11, 12, 14, 15, 16 undecahydrocyclopenta [a] phenanthren 3 one':ab,ti OR '11, 17 dihydroxy 17':ab,ti) AND '2 hydroxyacetyl':ab,ti AND '10, 13 dimethyl 2, 6, 7, 8, 9, 11, 12, 14, 15, 16 decahydro 1h cyclopenta [a] phenanthren 3 one':ab,ti OR '11beta, 17, 21 trihydroxypregn 4 ene 3, 20 dione':ab,ti OR '11beta, 17alpha, 21 trihydroxypregn 4 ene 3, 20 dione':ab,ti OR '14, 17 dihydroxy 14':ab,ti) AND '2 hydroxyacetyl':ab,ti AND '2, 15 dimethyltetracyclo [8.7.0.0':ab,ti AND '2, 7':ab,ti AND .0:ab,ti AND '11, 15':ab,ti AND '] heptadec 6 en 5 one':ab,ti OR '17 hydroxycorticosterone':ab,ti OR '4 pregnene 11beta, 17alpha, 21 triol 3, 20 dione':ab,ti OR '4 pregnene 3, 20 dione n beta, 17alpha, 21 triol':ab,ti OR 'acticort':ab,ti OR 'acticort 100':ab,ti OR 'aeroseb hc':ab,ti OR 'aeroseb-hc':ab,ti OR 'ala-cort':ab,ti OR 'ala-scalp':ab,ti OR 'ala-scalp hp':ab,ti OR 'alfacort':ab,ti OR 'algicortis':ab,ti OR 'alkindi':ab,ti OR 'alkindi sprinkle':ab,ti OR 'alpha derm':ab,ti OR 'alphaderm':ab,ti OR 'anucort-hc':ab,ti OR 'anumed-hc':ab,ti OR 'anutone-hc':ab,ti OR 'aquanil hc':ab,ti OR 'atrs 1902':ab,ti OR 'atrs1902':ab,ti OR 'balneol-hc':ab,ti OR 'barseb hc':ab,ti OR 'beta-hc':ab,ti OR 'biacort':ab,ti OR 'cetacort':ab,ti OR 'chronocort':ab,ti OR 'cobadex':ab,ti OR 'colocort':ab,ti OR 'compound f':ab,ti OR 'cordicare lotion':ab,ti OR 'coripen':ab,ti OR 'cort dome':ab,ti OR 'cort-dome':ab,ti OR 'cort-dome high potency':ab,ti OR 'cortef':ab,ti OR 'cortef cream':ab,ti OR 'cortenema':ab,ti OR 'cortibel':ab,ti OR 'corticorenol':ab,ti OR 'cortifan':ab,ti OR 'cortiphate':ab,ti OR 'cortisol':ab,ti OR 'cortisole':ab,ti OR 'cortispray':ab,ti OR 'cortoderm':ab,ti OR 'cortril':ab,ti OR 'cotacort':ab,ti OR 'covocort':ab,ti OR 'cremicort-h':ab,ti OR 'cutaderm':ab,ti OR 'derm-aid cream':ab,ti OR 'dermacrin hc lotion':ab,ti OR 'dermaid':ab,ti OR 'dermaid soft cream':ab,ti OR 'dermocare':ab,ti OR 'dermocortal':ab,ti OR 'dermolate':ab,ti OR 'dioderm':ab,ti OR 'eczacort':ab,ti OR 'ef cortelan':ab,ti OR 'efcortelan':ab,ti OR 'efmody':ab,ti OR 'egocort':ab,ti OR 'egocort cream':ab,ti OR 'eksalb':ab,ti OR 'eldecort':ab,ti OR 'emo-cort':ab,ti OR 'epicort':ab,ti OR 'ficortril':ab,ti OR 'filocot':ab,ti OR 'flexicort':ab,ti OR 'gly-cort':ab,ti OR 'glycort':ab,ti OR 'gynecort':ab,ti OR 'h-cort':ab,ti OR hc:ab,ti) AND hydrocortisone:ab,ti OR 'hc no. 1':ab,ti OR 'hc no. 4':ab,ti OR 'hebcort':ab,ti OR 'hebcort v':ab,ti OR 'hemorrhoidal hc':ab,ti OR 'hemril-30':ab,ti OR 'hemril-hc uniserts':ab,ti OR 'hi-cor':ab,ti OR 'hidrotisona':ab,ti OR 'hisone':ab,ti OR 'hycor':ab,ti OR 'hycort':ab,ti OR 'hydracort':ab,ti OR 'hydrasson':ab,ti OR 'hydro ricortex':ab,ti OR 'hydro-rx':ab,ti OR 'hydrocort':ab,ti OR 'hydrocorticosteroid':ab,ti OR 'hydrocortisate':ab,ti OR 'hydrocortison':ab,ti OR 'hydrocortisone 1% in absorbase':ab,ti OR 'hydrocortisone acetonide':ab,ti OR 'hydrocortisone astier':ab,ti OR 'hydrocortisone in absorbase':ab,ti OR 'hydrocortisone ointment':ab,ti OR 'hydrocortisone plus saline':ab,ti OR 'hydrocortisone steroid':ab,ti OR 'hydrocortisone, topical':ab,ti OR 'hydrocortisonum':ab,ti OR 'hydrocortisyl':ab,ti OR 'hydrocortone':ab,ti OR 'hydrogalen':ab,ti OR 'hydrokort':ab,ti OR 'hydrokortison':ab,ti OR 'hydrotopic':ab,ti OR 'hydventia':ab,ti OR 'hysone':ab,ti OR 'hytisone':ab,ti OR 'hytone':ab,ti OR 'hytone lotion':ab,ti OR 'incortin h':ab,ti OR 'infacort':ab,ti OR 'instacort 10':ab,ti OR 'kyypakkaus':ab,ti OR 'lacticare hc':ab,ti OR 'lacticare-hc':ab,ti OR 'lemnis fatty cream hc':ab,ti OR 'lenirit':ab,ti OR 'medihaler cort':ab,ti OR 'medihaler duo':ab,ti OR 'medrocil':ab,ti OR 'mildison':ab,ti OR 'mildison fet krem':ab,ti OR 'mildison lipocream':ab,ti OR 'mildison-fatty':ab,ti OR 'mitocortyl demangeaisons':ab,ti OR 'munitren':ab,ti OR 'nogenic hc':ab,ti OR 'novohydrocort':ab,ti OR 'nsc 10483':ab,ti OR 'nsc 741':ab,ti OR 'nsc10483':ab,ti OR 'nutracort':ab,ti OR 'optef':ab,ti OR 'otosone f':ab,ti OR 'penecort':ab,ti OR 'plenadren':ab,ti OR 'prepcort':ab,ti OR 'prevex hc':ab,ti OR 'procto-kit 1%':ab,ti OR 'procto-kit 2.5%':ab,ti OR 'proctocort':ab,ti OR 'proctosert hc':ab,ti OR 'proctosol-hc':ab,ti OR 'proctosone':ab,ti OR 'proctozone hc':ab,ti OR 'procutan':ab,ti OR 'rectasol-hc':ab,ti OR 'rectocort':ab,ti OR 'rederm':ab,ti OR 'sanatison':ab,ti OR 'scalp-aid':ab,ti OR 'schericur':ab,ti OR 'schericur 0.25%':ab,ti OR 'scherosone f':ab,ti OR 'sistral hydrocort':ab,ti OR 'skincalm':ab,ti OR 'stie-cort':ab,ti OR 'substance m':ab,ti OR 'synacort':ab,ti OR 'texacort':ab,ti OR 'triburon-hc':ab,ti OR 'unicort':ab,ti OR 'vasocort':ab,ti OR 'hydrocortisone':ab,ti 25965

#5 'suicide'/exp 73772

#6 'completed suicide':ab,ti OR 'self killing':ab,ti OR 'suicidal completion':ab,ti OR 'suicidal death':ab,ti OR 'suicidal fatality':ab,ti OR 'suicidal poisoning':ab,ti OR 'suicide death':ab,ti OR 'suicide, completed':ab,ti OR 'suicidium':ab,ti OR 'suicidum':ab,ti OR 'suicide':ab,ti 99794

#7 #1 OR #2 928729

#8 #3 OR #4 169521

#9 #5 OR #6 124006

#10 #7 AND #8 AND #9 334[Publication year from 1966 to 2024/09/04]

**China National Knowledge Infrastructure（CNKI） n=85**

(Topic: Depression Disorder) OR (Abstract or Keywords: Depression Disorder + Depression + Depressive Disorder + Depression Symptom Disorder + Depression) AND (Topic: Cortisol) OR (Abstract or Keywords: Hydrocortisone + Cortisol) AND (Topic: Suicide) OR (Abstract or Keywords: Suicide)

**Weipu Database（VIP） n=73**

(Topic = Depression + Depressive Disorder + Depression Disorder + Depression Symptom Disorder + Depression) AND (Topic = Hydrocortisone + Cortisol) AND (Topic = Suicide)

**Wanfang Database n=69**

(Topic: Depression Disorder) OR (Title or Keywords: Depression Disorder OR Depression OR Depressive Disorder OR Depression Symptom Disorder OR Depression) AND (Topic: Cortisol) OR (Title or Keywords: Hydrocortisone OR Cortisol) AND (Topic: Suicide) OR (Title or Keywords: Suicide)

# Supplementary Figures

**Supplementary Figure1**. Sensitivity analysis.

#
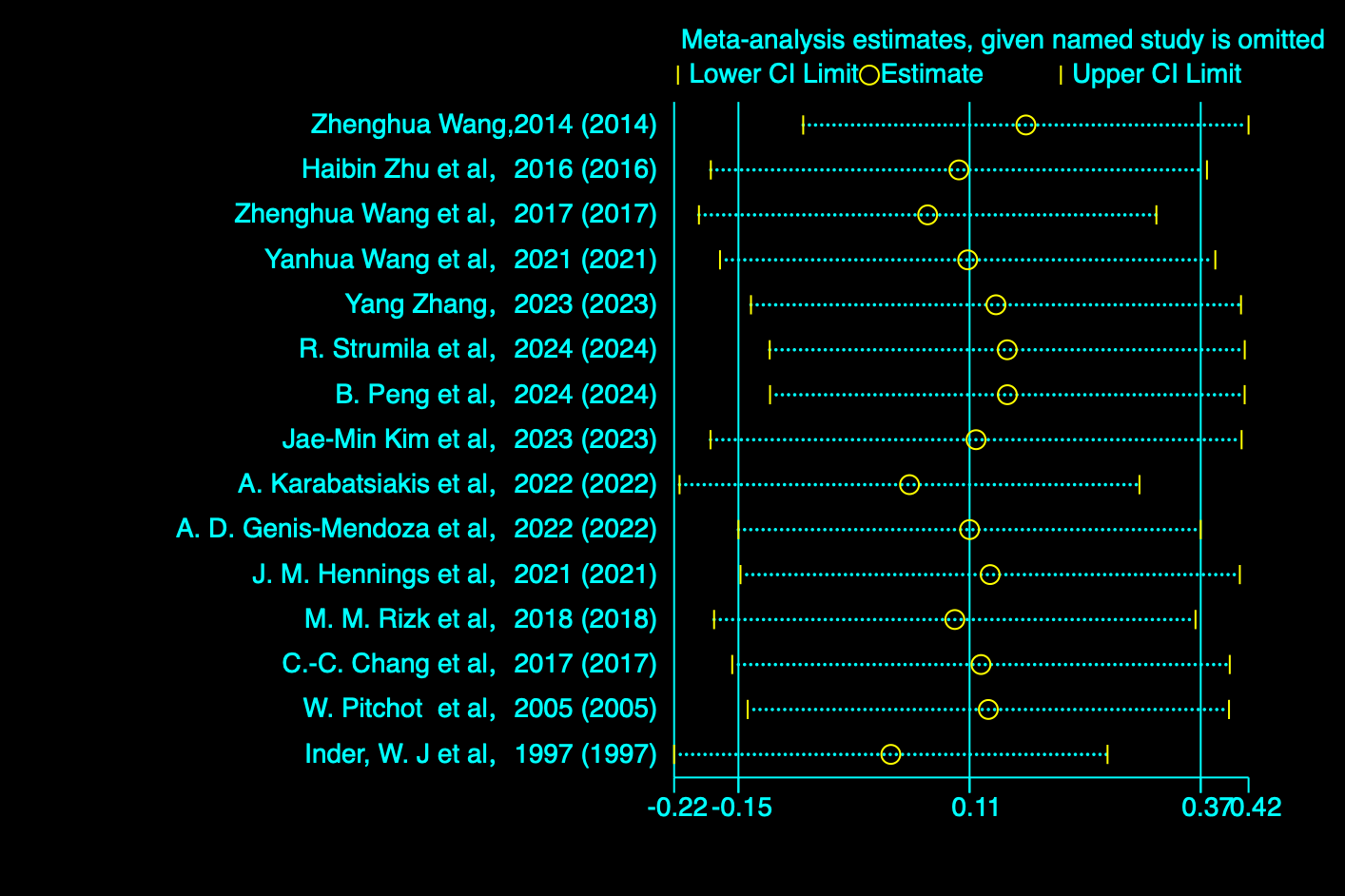


**Supplementary Figure2.** Funnel plot evaluating publication bias.


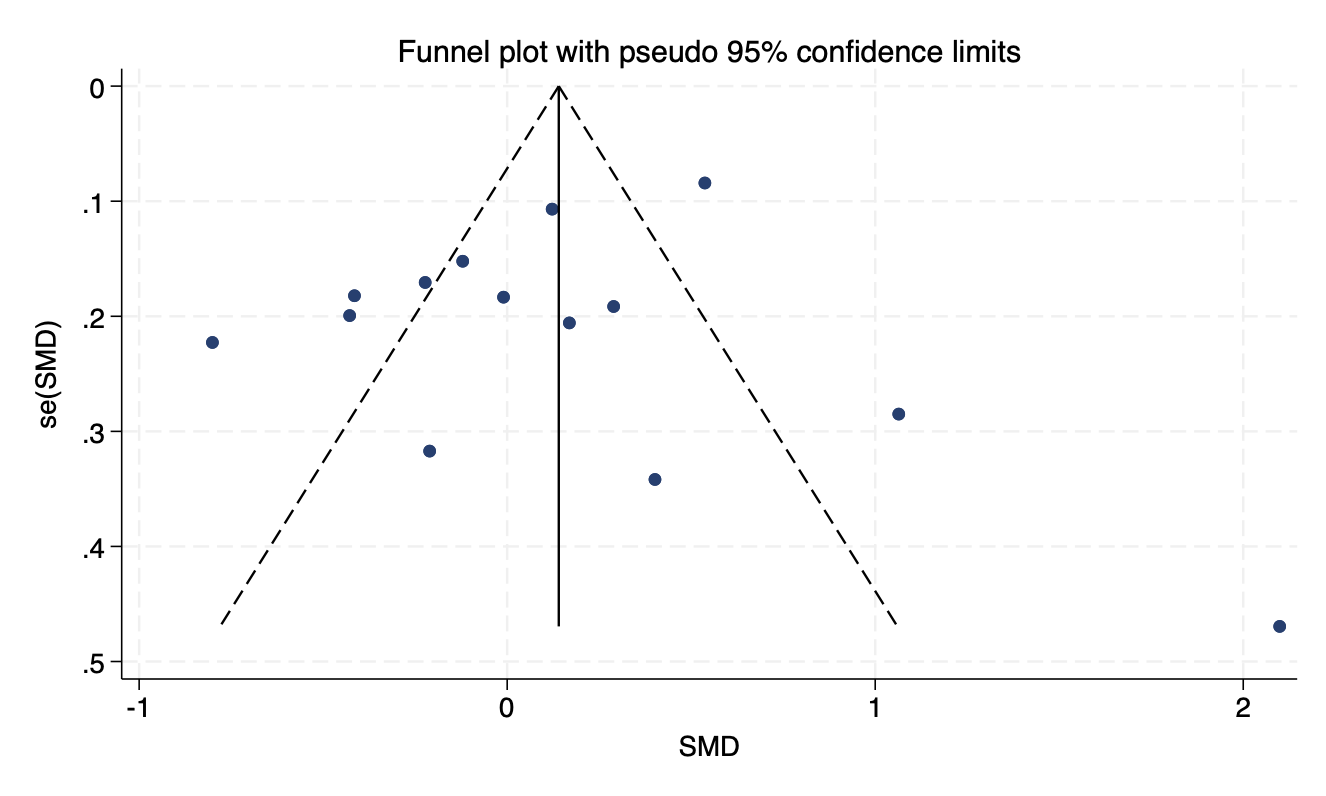


**
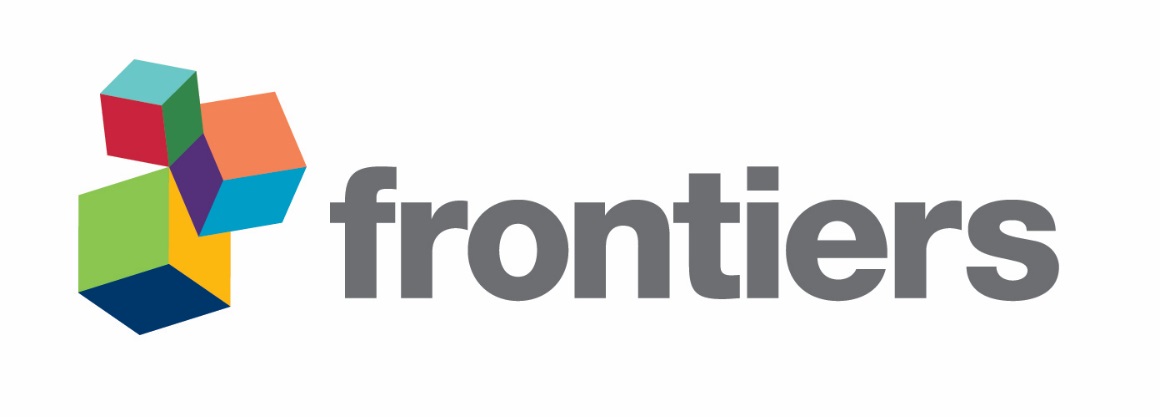
**
